# Sll1252 Coordinates Electron Transport between Plastoquinone and Cytochrome *b_6_/f* Complex in *Synechocystis* PCC 6803

**DOI:** 10.3390/genes14122151

**Published:** 2023-11-28

**Authors:** Radha Rani Balaga, Fumihiro Itoh, Suraj Chauhan, Mukulika Mandal, Pilla Sankara Krishna, Iwane Suzuki, Jogadhenu S. S. Prakash

**Affiliations:** 1 Department of Plant Sciences, School of Life Sciences, University of Hyderabad, Hyderabad 500046, India; balagaradha0404@gmail.com; 2 Graduate School of Life and Environmental Sciences, University of Tsukuba, Tennoudai 1-1-1, Tsukuba 305-8572, Japan; fumihiro_itoh@phytopetrum.com; 3 Department of Biotechnology and Bioinformatics, School of Life Sciences, University of Hyderabad, Hyderabad 500046, India; surajkchauhan9@uohyd.ac.in (S.C.); 20ltph07@uohyd.ac.in (M.M.); sankarakrishnahcu@gmail.com (P.S.K.); 4 Institute of Life and Environmental Sciences, University of Tsukuba, Tennoudai 1-1-1, Tsukuba 305-8572, Japan; iwanes6803@biol.tsukuba.ac.jp

**Keywords:** Sll1252, photosynthetic electron transport, suppressor mutant, PQ pool

## Abstract

A mutant, Δ*sll1252^ins^*, was generated to functionally characterize Sll1252. Δ*sll1252^ins^* exhibited a slow-growth phenotype at 70 µmol photons m^−2^ s^−1^ and glucose sensitivity. In Δ*sll1252^ins^*, the rate of PSII activity was not affected, whereas the whole chain electron transport activity was reduced by 45%. The inactivation of *sll1252* led to the upregulation of genes, which were earlier reported to be induced in DBMIB-treated wild-type, suggesting that Sll1252 may be involved in electron transfer from the reduced-PQ pool to Cyt *b_6_/f*. The inhibitory effect of DCMU on PSII activity was similar in both wild-type and Δ*sll1252^ins^*. However, the concentration of DBMIB for 50% inhibition of whole chain electron transport activity was 140 nM for Δ*sll1252^ins^* and 300 nM for wild-type, confirming the site of action of Sll1252. Moreover, the elevated level of the reduced-PQ pool in Δ*sll1252^ins^* supports that Sll1252 functions between the PQ pool and Cyt *b_6_/f*. Interestingly, we noticed that Δ*sll1252^ins^* reverted to wild-type phenotype by insertion of natural transposon, ISY523, at the disruption site. Δ*sll1252-N^trn^*, expressing only the C-terminal region of Sll1252, exhibited a slow-growth phenotype and disorganized thylakoid structure compared to wild-type and Δ*sll1252-C^trn^* (expressing only the N-terminal region). Collectively, our data suggest that Sll1252 regulates electron transfer between the PQ pool and the Cyt *b_6_/f* complex in the linear photosynthetic electron transport chain via coordinated function of both the N- and C-terminal regions of Sll1252.

## 1. Introduction

Cyanobacteria are primitive photosynthetic organisms and are considered to be ancestors of higher plant chloroplast [1,2,3,4,5]. Similarly, to the higher plants, the electron transport machinery, photosystem-II (PSII) and -I (PSI), are arranged in the thylakoid membranes. PSII comprises several polypeptides ranging from 3 to 60 KDa. Various proteomic studies and high-resolution electron microscopy [6,7,8,9,10] have helped identify more than 20 proteins associated with different cofactors that participate in redox reactions. Kashino et al. [6] reported the association of five novel proteins, Sll1252, Sll1130, Sll1390, Sll1414, and Sll1638, with highly purified PSII core complexes from the cyanobacterium *Synechocystis* PCC 6803 (hereafter *Synechocystis*). Subsequently, it was demonstrated that Sll1130 is not associated with the PSII complex; rather, it is a MazF toxin of a toxin–antitoxin system mediating heat-induced programmed cell death in *Synechocystis* [11]. Moreover, the Pakrasi group [6,12,13,14] worked with the remaining four novel polypeptides. Sll1638 is found to be a true homolog of higher plant PsbQ [12] along with PsbP, which modulates Ca^2+^ and Cl^−^ required for water splitting [15]. Sll1390 associates with the PSII complex to protect it from photodamage and oxidative stress, accelerating the repair mechanism [13], and Sll1414 is suggested to be involved in PSII biogenesis [14]. Sll1252 seems to be in the downstream of the signal transduction pathway and might sense the redox state of the plastoquinone (PQ) pool for balancing the electron flow between the photosystems [16]. The PQ pool in the thylakoid membrane relays electrons from PSII to PSI via the Cyt *b_6_/f* complex by reduction and re-oxidation. Cyanobacteria are known to coordinate the function of photosystems by sensing different light regimes and regulating the genes that code for proteins of photosynthetic complexes, thereby balancing the electron flow between the photosystems [17,18]. The functional coordination between the photosystems is maintained by regulating the stoichiometry of PSI/PSII [17,18,19]. Whole-transcriptome analysis under high light stress revealed that most of the PSII genes undergo transient downregulation at a short acclimation period except for the D1 protein, while the transcripts accumulate after prolonged exposure. In contrast, the downregulated expression of PSI genes implies a significant inflation of the PSII/PSI ratio during high light stress [19]. Subsequently, a small regulatory RNA PsrR1 has been demonstrated to post-transcriptionally regulate the expression of photosystem genes during high light-stress conditions [20]. The post-transcriptional regulatory mechanism describes a new mode of action to balance functional coordination between photosystems. In addition, mechanisms such as state transitions (due to the mobilization of phycobilisome and energy spillover) for rapid regulation of electron flow and balance between photosystems have also been reported [17,21,22,23,24,25].

The redox state of the PQ pool in turn regulates the expression of many genes of photosynthetic complexes to cope with irregularities and influences the downstream cellular processes [18,26,27,28]. However, the factors that sense the redox state of the PQ pool and balance the electron flow between the photosynthetic complexes are not yet completely understood. In this study, we have elucidated the function of an essential protein, Sll1252, as a regulator of electron transfer between the PQ pool and the Cyt *b_6_/f* complex in the linear photosynthetic electron transport in *Synechocystis*.

## 2. Materials and Methods

### 2.1. Cell and Culture Conditions

A glucose-tolerant strain of *Synechocystis* (wild-type) was initially obtained from Dr. J.G.K. Williams, Dupont de Nemours [29]. Wild-type cells were grown photo-autotrophically at 34 °C in BG-11 medium buffered with 20 mM HEPES-NaOH (pH 7.5), with a continuous supply of sterile air and illumination from incandescent lamps, as described previously [30]. Light intensities used for growth were 70 µmol photons m^−2^ s^−1^ (optimal light) or 5 µmol photons m^−2^ s^−1^ (low light). Cells were grown under continuous light illumination of 5 µmol photons m^−2^ s^−1^ in BG-11 media supplemented with 5 mM glucose for photo-mixotrophic growth, and 10 µM DCMU along with 5 mM glucose for photo-heterotrophic growth, respectively. Growth of the cultures was monitored by measuring OD at 730 nm using a UV-visible spectrophotometer (Model: UV-VIS 160A Shimadzu Co., Kyoto, Japan).

### 2.2. Insertional Inactivation of sll1252 by Transposon Mutagenesis (Δsll1252^ins^)

Δ*sll1252^ins^* mutant was generated by insertional inactivation of the gene, as described by Krishna et al. [31]. A DNA fragment containing the *sll1252* ORF with 80 bp upstream and 97 bp downstream flanking regions was PCR-amplified using primers *sll1252-F* and *sll1252-R* (Table A1). The resulting 957 bp fragment was ligated to the pT7Blue TA cloning vector using GeNei^TM^ instant cloning kit (Cat No: 107416, Bangalore Genei Pvt. Ltd., Bengaluru, India). The resultant plasmid, *pT-sll1252*, was used to inactivate the *sll1252* ORF by performing in vitro transposon reaction using the manufacturer’s instructions (EZ::Tn5^TM^ <KAN-2> Insertion kit, Cat No: EZ1982K, Epicentre, Madison, WI, USA). The plasmid DNA construct in which *sll1252* had been disrupted by kanamycin resistance gene cassette (*kan^r^*) was designated as *psll1252:: kan^r^*. This construct was used to transform the wild-type strain. The site of *kan^r^* insertion was located by sequencing *psll1252::kan^r^* using KAN-2 FP-1 primer (Table A1). The mutant strain thus generated was named Δ*sll1252^ins^*.

### 2.3. Screening for Spontaneous Suppressor Mutants of Δsll1252^ins^

Suppressor mutant colonies were isolated from Δ*sll1252^ins^* and streaked on BG-11 agar plates containing 5 mM glucose and kanamycin (25 µg per mL) under optimal light. After 2 to 3 weeks of incubation, selected colonies were grown in the liquid BG-11 media and genomic DNA was isolated. Specific primers Supp-F and Supp-R (Table A1) located at 146 bp upstream and 49 bp downstream to *sll1252*, respectively, were used for PCR. The resulting PCR-amplified product was sequenced using the kanamycin-specific primers KAN-2 FP-1 and KAN-2 RP-1 (Table A1).

### 2.4. Deletional Mutagenesis of the sll1252 Gene (Δsll1252^del^)

A 500 bp upstream and a 500 bp downstream flanking region of *sll1252* ORF from the genomic DNA were amplified using primer sets UF and UR; DF and DR, respectively. UF and DR primers contained *BglII* site. The PCR fragments thus generated using UF-UR and DF-DR primer sets were used to set up a fusion PCR with the UF-DR primer set. The fusion PCR-amplified product thus generated was cloned into pMD19 T-Vector (Takara-Bio., Otsu, Japan). The resultant construct was named *p*T-*sll1252^del^*. *kan^r^* was amplified using the primers containing *SalI* restriction site: KAN-2 SalI-F and KAN-2 SalI-R (Table A1). *SalI-*digested Kanamycin gene was cloned onto the *BglII* site of the *p*T-*sll1252^del^* by blunting followed by ligation. The mutant was generated by natural transformation in wild-type cells and named Δ*sll1252^del^.*

### 2.5. Construction of sll1252 N-Terminal Truncated Mutant (Δsll1252-N^trn^)

A 500 bp upstream flanking region and 327 bp toward the end of the *sll1252* ORF covering the S4-domain were amplified from the genomic DNA using primer sets UF and *sll1252-trn*-UR; *sll1252-N^trn^*-F and *sll1252-N^trn^-R*, respectively. *sll1252-N^trn^-F* primer was designed with 15 nucleotides complementary to the 5′ region of *sll1252-trn-UR* primer, followed by ATG. A *smaI* site was added in both primers. The PCR fragments thus generated using UF, *sll1252-trn*-UR and *sll1252-N^trn^-F*, *sll1252-N^trn^-R* primer sets were used to set up a fusion PCR with UF and *sll1252-N^trn^-R* primer set. A second fusion PCR was performed using *sll1252-N^trn^-DF* and DR primer sets. The fusion PCR-amplified product thus generated was cloned in pMD19 cloning vector (Takara Inc., Kyoto, Japan). The resultant construct was named *p*T-*sll1252-N^trn^.* The SalI-digested *kan^r^*, as described in the previous section, was cloned at the *smaI* site of *p*T-*sll1252-N^trn^* by blunting followed by ligation. The final construct, in which first 456 bases of the *sll1252* ORF had been deleted, was transformed to *Synechocystis*. The *Synechocystis* strain thus generated was named Δ*sll1252-N^trn^.*

### 2.6. Construction of sll1252 C-Terminal Truncated Mutant (Δsll1252-C^trn^)

A 953 bp DNA fragment including 500 bp upstream flanking region, 453 bp *sll1252* ORF, and 500 bp downstream flanking region were amplified from the genomic DNA using primer sets UF, *sll1252-trn*-DR and *sll1252-C^trn^*-F, DR, respectively. *sll1252-trn*-DR primer was designed with 14 nucleotides complementary to 5′ region of *sll1252-C^trn^*-F primer along with a stop codon (TAA). *smaI* site was added to *sll1252-trn-DR* and *sll1252-C^trn^-F* primers. The PCR fragments generated were used to set up a fusion PCR with UF and DR primer sets. The fusion PCR-amplified product thus generated was cloned into pMD19 T-Vector (Takara-Bio). The resultant construct was named *p*T-*sll1252-C^trn^*. As mentioned above, *kan^r^* was cloned into *smaI* site of *p*T-*sll1252-C^trn^* construct by blunting followed by ligation. Thus, the final construct, in which the 327 bp toward the end of the *sll1252* ORF had been deleted, was used to transform wild-type cells of *Synechocystis*. The *Synechocystis* strain thus generated was named Δ*sll1252-C^trn^.*

### 2.7. Measurement of Photosynthetic Evolution of Oxygen

Photosynthetic oxygen evolution was measured in 1 mL of cell suspension (OD_730_ of ~1, approximately 5 µg/mL chlorophyll), using an oxygen electrode (Oxygraph plus, Hansatech Instruments Ltd., Norfolk, England). Wild-type and Δ*sll1252^ins^* were cultivated photo-autotrophically under optimal or low light with continuous aeration. The rates of whole chain electron transport (WCE) activity and PSII-catalyzed electron transport activity were determined in terms of oxygen evolution in the presence of 2 mM Methyl viologen and 1.0 mM phenyl-*p-*benzoquinone (PBQ), respectively, under illumination of 1000 µmol photons m^−2^ s^−1^. The activity was determined as a difference between net oxygen evolution in light and oxygen consumption in darkness. The effect of 2,5-dibromo-3-methyl-6-isopropyl-*p-*benzoquinone (DBMIB) at increasing concentrations (0 to 800 nM) was analyzed by measuring the oxygen-evolving activity in the presence of 2 mM Methyl viologen. The rate of PSII-catalyzed electron transport was measured in the presence of increasing concentrations (0 to 600 nM) of 3-(3,4-dichlorophenyl)-1,1-dimethylurea (DCMU). The chlorophyll concentration of cultures was determined using 80% acetone as a solvent for extraction [32].

### 2.8. Preparation of cDNA for DNA Microarray Analysis

Wild-type and Δ*sll1252^ins^* cells, grown under optimal and low light, were killed instantaneously by the addition of 50 mL ice-cold phenol/ethanol (1:20, *w*/*v*) to 50 mL of the cell suspension and then total RNA was extracted, as described previously [30]. The RNA was treated with DNase I (Cat no. 314-08071; Nippon Gene, Tokyo, Japan) to remove contaminating DNA. cDNAs, labeled with fluorescent dyes (Cy3 and Cy5; GE Health Care, Tokyo, Japan), were prepared from 10 μg total RNA with an RNA Fluorescence Labeling Core kit (Cat no. TX807, M-MLV, version 2.0; Takara-Bio) according to the manufacturer’s instructions.

### 2.9. DNA Microarray Analysis

Genome-wide analysis of transcript levels was performed with DNA microarrays, as described previously [30]. In brief, we used a DNA microarray chip (CyanoCHIP; Takara-Bio) that covered 3079 of the 3168 open-reading frames (97% of all genes, except transposon-related genes) of the *Synechocystis* genome. Hybridization of labeled cDNAs to the DNA microarray probes was allowed to proceed at 65 °C for 16 h. After hybridization, the microarray chips were rinsed with 2x SSC (1x SSC is 150 mM NaCl and 15 mM sodium citrate) at room temperature. They were washed with 2x SSC at 60 °C for 10 min and with 0.2x SSC, 0.1% SDS at 60 °C for 10 min and then rinsed with distilled water at room temperature for 2 min. Moisture was removed with an air spray before analysis with the array scanner (Cat no. GMS418; Affymetrix, Woburn, MA, USA). Each signal was quantified with the ImaGene ver. 6.0 program (BioDiscovery, Los Angeles, CA, USA). The signal from each gene on the microarray was normalized by reference to the total intensity of signals from all genes, with the exception of 159 genes for rRNAs. We then calculated the change in the level of the transcript of each gene relative to the total amount of mRNA.

### 2.10. Transmission Electron Microscopy

Transmission electron microscopy (TEM) was performed according to Yubuki et al. [33] with few modifications. Cell suspensions were mixed with an equal volume of 1% glutaraldehyde in sodium cacodylate buffer (0.2 M, pH 7.2) and incubated at 4 °C for 2 h. After washing with a cacodylate buffer, the cells were fixed by incubation in 1% osmium tetroxide at 4 °C for 2 h. The cells were dehydrated through a series of ethanol concentrations: 50% ethanol for 1 h, followed by 75, 90, 95, and 99.5% ethanol for 30 min each. Dehydration was followed by treatment of cells with a 1:1 mixture of 99.5% ethanol and propylene oxide twice for 10 min each and then with 99.5% propylene oxide twice for 10 min. The propylene oxide was replaced with AGAR Low viscosity resin (Cat no: R1370, Agar Scientific Ltd., Stansted, England) by treating with a 1:1 mixture of propylene oxide and resin, followed by treatment with the resin. The resin was polymerized at 70 °C for 12 h. Thin sections (50 nm thickness) were cut with an ultramicrotome (EM-Ultracuts, Reichert, Germany) and stained with 2% uranyl acetate and lead citrate. The samples were observed under a transmission electron microscope (H-7650, Hitachi, Tokyo, Japan).

## 3. Results

### 3.1. Sll1252 Is a Conserved S4-Domain Containing Protein

**Figure 1:**
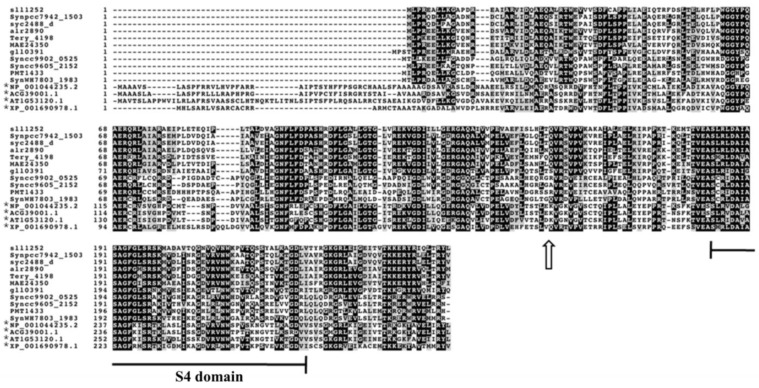
Multiple sequence alignment of the Sll1252 from *Synechocystis* sp. PCC 6803 and its homologous proteins in other cyanobacterial genomes, algae and higher plants. The sequences were aligned by the ClustalW algorithm. Identical amino acids are shown in black and conservative substitutions are in gray. * indicates algae and higher plants. *NP_001044235.2*, *Oryza sativa*; *ACG39001.1*, *Zea mays*; *AT1G53120.1*, *Arabidopsis thaliana*; *XP_001690978.1*, *Chlamydomonas reinhardtii*; *Synpcc7942_1503*, *Synechococcus elongatus PCC7942*; *syc2488_d*, *Synechococcus elongatus PCC6301*; *alr2890*, *Anabaena* sp.*PCC7120*; *Tery_4198*, *Trichodesmium erythraeum IMS101*; *MAE24350*, *Microcystis aeruginosa NIES-843*; *sll1252*, *Synechocystis* sp. *PCC6803*; *gll0391*, *Gloeobacter violaceus PCC7421*; *Syncc9902_0525*, *Synechococcus* sp. *CC9902*; *Syncc9605_2152*, *Synechococcus* sp. *CC9605*; *PMT1433*, *Prochlorococcus marinus MIT9313*; *SynWH7803_1983*, *Synechococcus* sp. *WH 7803*. The S4-domain is labeled. The site of *kan^r^* insertion in Δ*sll1252^ins^* is indicated with an open arrow.

Sll1252 protein is shown to have important physiological functions in *Synechocystis* and is conserved both in cyanobacteria and higher plants. It is associated with photosystem II and is suggested to be involved in regulating photosynthetic electron transport [6,16]. In this study, we generated a mutant of *sll1252* by insertional inactivation and subsequently characterized the resultant mutant (Δ*sll1252^ins^*) phenotypically to obtain deeper insights into its physiological function. The protein has a calculated mass of 28.4 kDa. Computational domain analysis indicated an S4-like RNA binding domain in the C-terminal region of Sll1252, suggesting it to be a probable RNA binding protein (Figure 1).

### 3.2. Mutagenesis of sll1252 in Synechocystis

**Figure 2:**
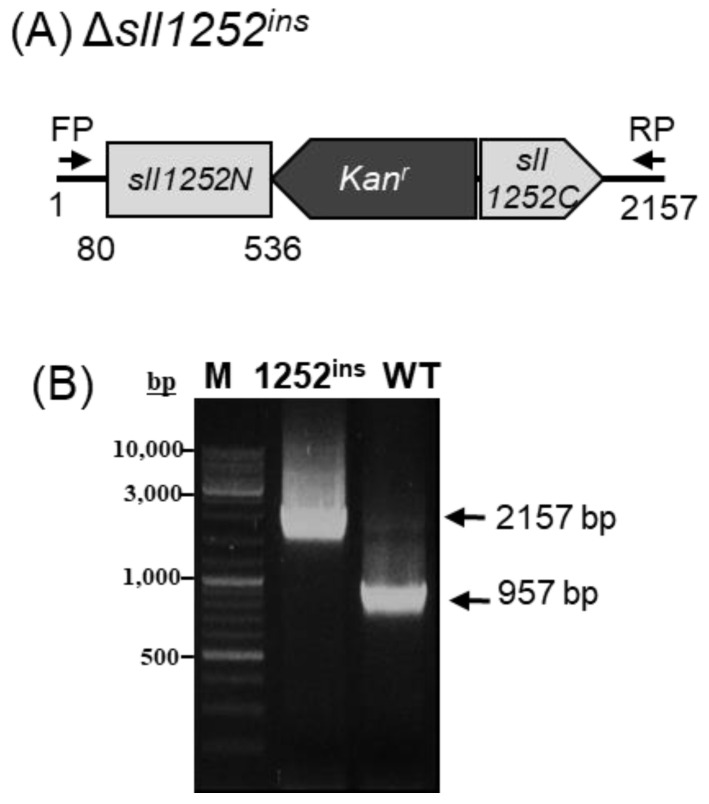
Strategy for disruption of *sll1252* gene in the genome of *Synechocystis* sp. PCC 6803. The wild-type copy of the *sll1252* gene was completely replaced by ∆*sll1252^ins^*. (**A**) A 957 bp DNA fragment corresponding to *sll1252* was insertionally inactivated *kan^r^* (1200 bp). (**B**) Schematic representation of the genotype of ∆*sll1252^ins^*. The *sll1252* and *kan^r^* cassette are shown in light gray and dark gray arrows, respectively. Thick arrows indicate *sll1252-F* (FP) and *sll1252-R* (RP) primers used for PCR amplification of the wild-type copy of *sll1252* gene and that of the *kan^r^* cassette. M represents 1 kb DNA ladder; WT, PCR product with wild-type DNA as template; *1252^ins^*, PCR product with Δ*sll1252^ins^* DNA as template.

To elucidate the function of Sll1252, the ORF, *sll1252*, was disrupted by inserting a kanamycin resistance gene by in vitro transposon mutagenesis, described in the Materials and Methods (Figure 2A). The *kan^r^* was inserted within the *sll1252* ORF, as shown in the schematic representation (Figure 2A). As *Synechocystis* contains multiple copies of identical genomes, a mutant without functional Sll1252 can be obtained only after the replacement of all copies of *sll1252* by the inactivated *sll1252.* The extent of replacement of wild-type copies of *sll1252* by the mutant copies was analyzed by PCR amplification, which indicated complete segregation of the mutated copy. After several rounds of culturing the mutant cells in increasing concentrations of kanamycin, the genomic DNA was isolated to check the segregation. With the genomic DNA from wild-type cells as a template, a fragment of 957 bp corresponding to the *sll1252* ORF and its upstream and downstream flanking regions was amplified by PCR. With the same set of primers, a 2157 bp DNA fragment, corresponding to the wild-type fragment (957 bp) with the inserted kanamycin gene (1200 bp), was amplified when the genomic DNA of Δ*sll1252^ins^* cells was used as a template (Figure 2B). These observations suggest that the *sll1252* ORF in all genomes was completely disrupted by the kanamycin gene cassette in the Δ*sll1252^ins^* strain.

### 3.3. Insertional Inactivation of sll1252 Influences Growth at Optimal Light

**Figure 3:**
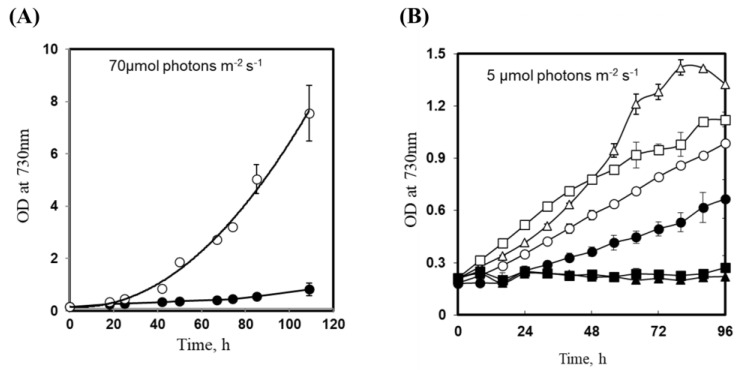
Effect of light intensity on growth profiles of wild-type and Δ*sll1252^ins^*. (**A**) Photo-autotrophic growth of wild-type (open circles) and Δ*sll1252^ins^* (closed circles) at optimal light. (**B**) Photo-autotrophic growth of wild-type (open circles) and Δ*sll1252^ins^* (closed circles) at low light. Photo-heterotrophic growth in the presence of 5 mM glucose and 10 µM of DCMU of wild-type (open triangles) and Δ*sll1252^ins^* (closed triangles). Growth under mixotrophic conditions (5 mM glucose) of wild-type (open squares) and Δ*sll1252^ins^* (closed squares). Standard deviations are shown in vertical bars. h, hours.

To analyze whether the disruption of *sll1252* would influence the physiological activities of the cells, we compared the growth profiles of wild-type and Δ*sll1252^ins^* under optimal light. As shown in Figure 3A, the growth of Δ*sll1252^ins^* cells was much slower than wild-type cells. Insertional inactivation and slow-growth phenotype of Δ*sll1252^ins^* demonstrated that Sll1252 plays an essential role in the physiology of the cells. As Sll1252 seems to be associated with photosystem II [6,16], the mutation in *sll1252* might have influenced the photosynthetic efficiency and, thereby, caused slow-growth phenotype. Hence, we compared the growth of wild-type and Δ*sll1252^ins^* strains at low light under photo-autotrophic, photo-heterotrophic, and photo-mixotrophic conditions. As shown in Figure 3B, Δ*sll1252^ins^* mutant exhibited slow-growth phenotype under photo-autotrophic conditions at low light. Under photo-heterotrophic and -mixotrophic conditions, growth was not detected in Δ*sll1252^ins^* culture compared to wild-type (Figure 3B). This suggests that the insertional inactivation of *sll1252* leads to a light-sensitive and glucose-sensitive phenotype.

### 3.4. Insertional Inactivation of sll1252 Alters the Rate of Whole Chain Electron Transport

The rate of PSII-catalyzed electron transport and WCE activity was compared in the wild-type and Δ*sll1252^in^^s^* to elucidate the effect of inactivation of *sll1252* on photosynthesis. As shown in Table 1, the rate of WCE in Δ*sll1252^ins^* cells was reduced by 45% compared to wild-type cells.

### 3.5. The Global Gene Expression Profiles of DBMIB-Treated Wild-Type and Δsll1252^ins^ Cells Grown under Optimal Light Are Similar

**Figure 4:**
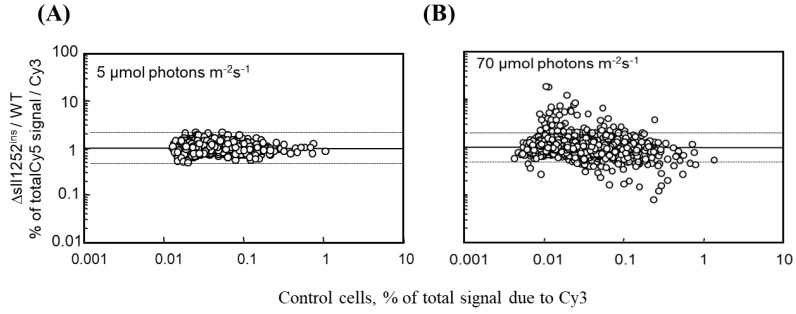
DNA microarray analysis to compare the gene expression changes in ∆*sll1252^ins^* and wild-type cells. RNA extracted from wild-type and ∆*sll1252^ins^* cells was used to synthesize Cy3- and Cy-5-labeled cDNAs, respectively. (**A**) Cy5/Cy3 ratio of almost all the genes was between 2.0 and 0.5 (indicated by dashed lines), implying no alteration in the gene expression profile at low light. (**B**) Cy5/Cy3 ratio of several genes were altered in Δ*sll1252^ins^* when grown at optimal light, suggesting that the insertional inactivation of *sll1252* affected the gene expression. Similar results were obtained in two independent experiments, and the figure represents one of the experiments.

Since the photosynthetic transfer of electrons and/or the redox status of electron-transport components are the primary signals for the expression of few genes, we investigated the effect of *sll1252* insertional inactivation on the genome-wide expression of genes in Δ*sll1252^ins^* and wild-type grown under low light (Figure 4A). It was observed that the insertional inactivation did not affect the global gene expression profile compared to wild-type at low light (Figure 4A). However, we observed that the expression of 37 genes was upregulated more than 2.5 times in Δ*sll1252^ins^* compared to wild-type, when grown under optimal light (Figure 4B). Genes that were upregulated in Δ*sll1252^ins^* are listed in Table 2. It was observed that 7 out 37 upregulated genes in Δ*sll1252^ins^* were also reported to be commonly upregulated in DBMIB- or DCMU-treated wild-type cells. Fifteen upregulated genes were reported to be exclusively induced in the wild-type cells with DBMIB, but none of the upregulated genes were reported to be exclusively induced by DCMU [18] (Table 2). Thus, the effect of *sll1252* insertional inactivation is similar to the inhibitory effect of DBMIB on global gene expression profile. This indicates that Sll1252 is involved in the electron transfer from the reduced-PQ pool to the Cyt b_6_/*f* complex. With this clue, to establish the involvement of Sll1252 in the regulation of electron transport, we analyzed the relative sensitivity of wild-type and Δ*sll1252^ins^* to DCMU, which act as an inhibitor of electron transfer between PSII and Q_B_. Similarly, the relative sensitivity to DBMIB, which inhibits electron transfer between the PQ pool and Cyt b_6_/*f*, was also analyzed.

### 3.6. Δsll1252^ins^ Cells Are More Sensitive to Inhibition by DBMIB Than Wild-Type Cells

**Figure 5:**
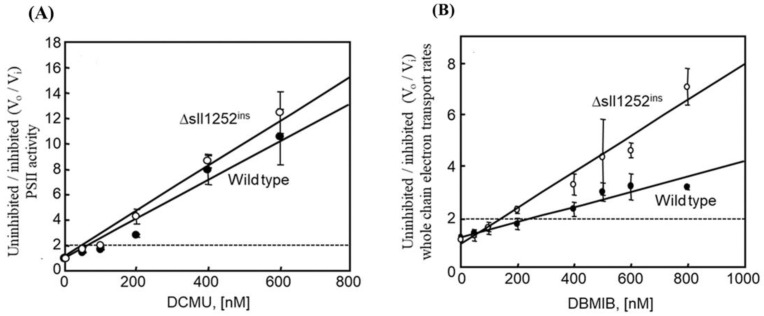
Effect of varying concentrations of DCMU and DBMIB on photosystem II and whole chain electron transport, respectively. Rates of uninhibited/inhibited (V_0_/V_i_) activities plotted as a function of DCMU (**A**) and DBMIB (**B**) concentrations. Three independent experiments were performed, and the data are represented as mean ± SD: wild-type cells (filled circles) and Δ*sll1252^ins^* cells (open circles). Standard deviations are shown as vertical bars.

Changes in the DNA microarray gene expression profile and the decrease in the WCE activity of ∆*sll1252^ins^* suggest that Sll1252 is required for electron transfer from the PQ pool to the Cyt *b_6_/f* complex. Differential sensitivity of wild-type and Δ*sll1252^ins^* cells to specific inhibitors, DCMU and DBMIB, was examined by measuring photosynthetic electron transport rates at increasing concentrations of the inhibitors. The oxygen-evolving activity with PBQ as an electron acceptor under the rate-saturating irradiance showed a gradual decrease in PSII activity with an increase in the concentration of DCMU in both wild-type and Δ*sll1252^ins^* (Figure 5A). The ratios of uninhibited (V_0_)/inhibited (V_i_) PBQ-mediated PSII activities versus DCMU concentration were calculated. The DCMU required for 50% inhibition of the original activity was almost the same (80 nM) in both wild-type and Δ*sll1252^ins^* cells. These results suggest that the mutation in *sll1252* had no effect on the site of DCMU action and confirm that the Sll1252 is not involved in the PSII-catalyzed electron transport activity. We analyzed the effect of increasing concentrations of DBMIB on WCE activity in the wild-type and Δ*sll1252^ins^* cells with Methyl viologen as an electron acceptor (Figure 5B). The inhibitor required for 50% inhibition of the WCE activity was 300 nM in the wild-type and 140 nM in Δ*sll1252^ins^* cells. Moreover, Δ*sll1252^ins^* had elevated levels of reduced-PQ pool compared to wild-type. The reduced-PQ content in Δ*sll1252^ins^* is almost similar to DBMIB (an inhibitor which blocks electron transport between the PQ pool and the Cyt *b_6_/f* complex)-treated wild-type cells (Figure S1), which suggests that the site of action of Sll1252 is between the PQ pool and Cyt *b_6_/f*. This suggests that the Δ*sll1252^ins^* cells are more sensitive to DBMIB and confirms that Sll1252 is required for electron transport from the PQ pool to the Cyt *b_6_/f* complex.

### 3.7. Suppressor Mutants of Δsll1252^ins^ (Δsll1252^ins^-Rev)

**Figure 6:**
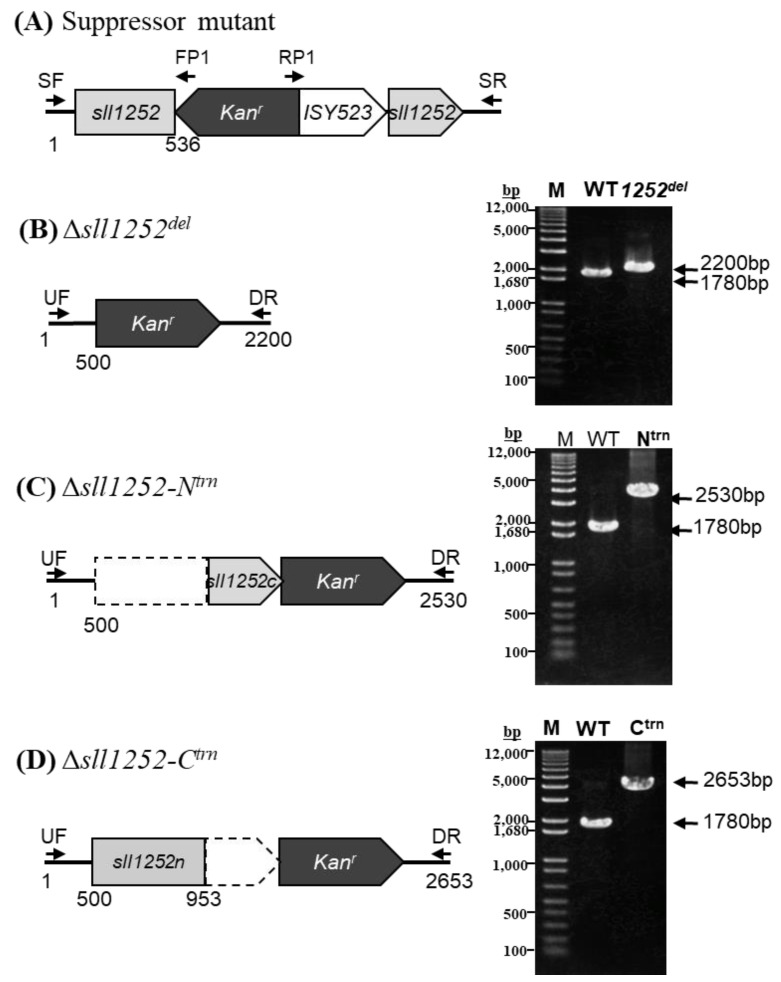
(**A**) Schematic representation of the genotype of ∆*sll1252^ins^-Rev*. Insertional inactivation of *sll1252* was suppressed by spontaneous insertion of naturally occurring transposon, ISY523. An open arrow indicates ISY523. Supp-F (SF) and Supp-R (SR) primers are used to amplify DNA fragments covering the *sll1252* ORF. KAN-2 FP-1 (FP1) and KAN-2 RP-1 (RP1) kanamycin-specific primers were used for sequencing to locate the position of spontaneous insertion of ISY523. (**B**) Schematic representation of the genotype of Δ*sll1252^del^* cells. A 1780 bp DNA fragment corresponding to *sll1252* ORF was replaced with *kan^r^* (1200 bp). PCR analysis with the primers as indicated in the construct map; M represents 1 kb plus DNA ladder; WT, PCR product with wild-type DNA as template; *1252^del^*, PCR product with Δ*sll1252^del^* DNA as template. (**C**) Schematic representation of the genotype of Δ*sll1252-N^trn^*. A 453 bp DNA fragment corresponding to 5′ region of *sll1252* was deleted using fusion PCR, as described in Materials and Methods. PCR analysis with the primers as indicated in the construct map; WT, PCR product with wild-type DNA as template; *N^trn^*, PCR product with Δ*sll1252-N^trn^* DNA as template. (**D**) Schematic representation of the genotype of the Δ*sll1252-C^trn^* mutant. A 327 bp DNA fragment corresponding to 3′ region of the *sll1252* gene was deleted using fusion PCR. PCR analysis with the primers as indicated in the construct map; WT, PCR product with wild-type DNA as template; *C^trn^*, PCR product with Δ*sll1252-C^trn^* DNA as template. Thick arrows in panels B, C, and D indicate *UF* and *DR* primers used for PCR amplification of the wild-type and mutant copy including *kan^r^* cassette. *sll1252* and *kan^r^* are shown in light gray and dark gray arrows, respectively. The deleted part of *sll1252* is shown with dotted lines in Δ*sll1252-N^trn^* and Δ*sll1252-C^trn^* construct maps.

Few Δ*sll1252^ins^* colonies grew faster than others with different colony morphology in the BG-11 agar plates containing kanamycin. Hence, we analyzed the kanamycin insertion site of the phenotypically different colonies by PCR amplification and DNA sequencing. Surprisingly, we observed the presence of a naturally occurring cyanobacterial transposable element (ISY523) along with *kan^r^* used for the inactivation of the *sll1252* at the insertion site (Figure 6A). The appearance of transposable elements at the inactivation site caused the cells to revert to the wild-type phenotype, labeled as Δ*sll1252^ins^-Rev*. Reversion of the mutant to the wild-type phenotype suggests that the insertional inactivation of *sll1252* might be detrimental to the cells.

### 3.8. Truncated and Deletional Mutants of sll1252 (Δsll1252N^trn^, Δsll1252C^trn^, and Δsll1252^del^)

The mean generation time calculated for Δ*sll1252^del^* (Figure 6B) is 9.8 h with no effect on PSII and WCE activities compared to wild-type (Table 1). We assumed that in Δ*sll1252^ins^*, the insertion of *kan^r^* might have resulted in the expression of either the N-terminal or C-terminal region of Sll1252 (Sll1252-N or Sll1252-C), leading to the severe slow-growth phenotype. The natural transposon has been placed between the kanamycin and C-terminal encoding region of *sll1252*, probably to inhibit the expression of either Sll1252-N or Sll1252-C, thereby suppressing the mutation effect. Thus, the expression of either Sll1252-N or Sll1252-C may be detrimental to the cells. To test this hypothesis, two truncated mutants were generated: Δ*sll1252-N^trn^* expressing the N-terminal region consisting of 453 bp from start codon and Δ*sll1252C^trn^* expressing the C-terminal region covering 454 bp till the end of the *sll1252* ORF (Figure 6C,D). The constructs thus generated were transformed into wild-type cells. Complete replacement of the wild-type copies of *sll1252*, by Δ*sll1252-N^trn^* or Δ*sll1252-C^trn^*, were confirmed by PCR amplification using UF and DR primer (Figure 6C,D, Table A1).

### 3.9. Expression of Only C-Terminal Region of sll1252 Influences Growth of the Cells

As expected, we observed no significant decrease in the photosynthetic oxygen evolution in Δ*sll1252^ins^-Rev*, Δ*sll1252^del^*, and Δ*sll1252C^trn^*. In Δ*sll1252N^trn^* cells, there was a 50% decrease in the WCE rate (Table 1). A slight decline in PSII-catalyzed oxygen evolution was observed in all strains of *sll1252* mutants, except for Δ*sll1252C^trn^*, in which approximately 40% loss in the PSII activity was observed (Table 1). The mean generation time calculated for wild-type cells was ~8 h and for Δ*sll1252^del^* it was 9.8 h. As mentioned before, Δ*sll1252^ins^* exhibited a severe slow-growth phenotype. The mean generation time of Δ*sll1252-N^trn^* was 22 h. Since only Δ*sll1252-N^trn^* exhibited slow-growth phenotype like Δ*sll1252^ins^* and reduction in whole chain electron transport, it suggests that expression of the C-terminal region of Sll1252 is deteriorative to the cells (Table 1). Hence, we hypothesize that both the C-terminal and N-terminal regions of Sll1252 regulate the electron transport between the plastoquinone pool and the Cyt *b_6_/f* complex.

### 3.10. Thylakoid Membrane Is Disorganized in Δsll1252N^trn^

**Figure 7:**
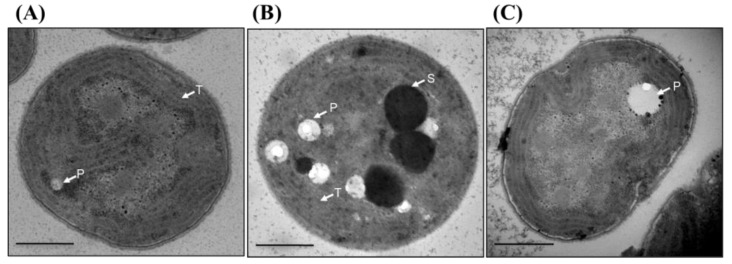
Changes in cell morphology due to truncation of 5′ and 3′ regions of the *sll1252* ORF. Representative electron micrograph of (**A**) wild-type cells, (**B**) Δ*sll1252-N^trn^*, and (**C**) Δ*sll1252*-*C^trn^* cells. The cells were grown at optimal light for 24 h and then fixed for transmission electron microscopic observations. Carboxysomes (C), thylakoid membrane (T), and polyphosphate bodies (P) are indicated by arrows. Black spherical bodies (S) are seen only in the Δ*sll1252-N^trn^* mutant. Scale bar = 500 nm.

Since we observed slow-growth phenotype in Δ*sll1252-N^trn^*, but not in Δ*sll1252-C^trn^*, we further examined the ultrastructural changes caused in cells due to expression of the truncated forms of Sll1252. The wild-type and Δ*sll1252-C^trn^* looked alike, as can be seen in Figure 7. Well-organized thylakoid membranes are visible when examined under transmission electron microscope. Layers of thylakoid membranes were arranged in rows. In contrast to wild-type and Δ*sll1252-C^trn^* cells, thylakoid membranes were disorganized in Δ*sll1252-N^trn^*. Only a few polyphosphate granules were observed in wild-type and Δ*sll1252-C^trn^* cells, indicating actively growing and dividing cells in these cultures, which might be using the stored phosphate for their active growth. The presence of more polyphosphates in Δ*sll1252-N^trn^* might be a reason for its slow-growth phenotype. The microscopic image revealed spherical dense bodies in Δ*sll1252-N^trn^*, probably containing the degraded thylakoid membrane lipids. Thus, it is clear from the transmission electron microscopic observations that expression of only the C-terminal region of Sll1252 leads to ultrastructural changes, especially the disorganization of thylakoid membranes. The observations thus support the essential physiological role of Sll1252 in photosynthetic electron transport.

## 4. Discussion

In the present study, using DNA microarray gene expression profiling and comparative transcriptome analysis, we can obtain clues about the function of Sll1252. Sll1252, associated with the thylakoid membrane, is now proven to be an essential protein in photosynthetic electron transport, assisting election transfer between the PQ pool and the Cyt *b_6_/f* complex. The genes upregulated due to insertional inactivation of *sll1252* (Table 1) are reportedly induced by DBMIB, which inhibits electron transfer from the PQ pool to the Cyt *b_6_/f* complex. Physiologically, ∆*sll1252^ins^* had a slow-growth phenotype and decreased WCE activity (Figure 3, Table 1). The elevated level of the reduced-PQ pool in ∆*sll1252^ins^* suggests that Sll1252 might facilitate electron transfer from the PQ pool to the Cyt *b_6_/f* complex (Figure S1). This is consistent with the observation by Kashino et al. [16]. The insertionally disrupted mutant is observed to be reverted by mobilization of a naturally occurring transposable element (ISY523) by an unknown mechanism. Interestingly, *Synechocystis* cells use a natural transposable element to suppress the mutations that occur in genes, if they are lethal to the cells [35]. The mechanism by which these cells place the transposable element at the insertion site to suppress the mutation is yet to be elucidated. It is also interesting to study whether the placement of the transposon for suppressing the lethal effect of the mutation is by selective insertion or random transposition.

Further, Δ*sll1252^ins^* also exhibited glucose sensitivity. Earlier studies, using comparative genome analysis of *Synechocystis*, revealed that frameshift/substitution mutations in several genes, like glcP encoding glucose transporter and the genes encoding protein components of NDH-complexes involved in respiratory electron transport, result in glucose sensitivity [36,37]. Moreover, inactivation of *pmgA*, which is known to be essential for photo-mixotrophic growth and involved in the regulation of photosystem stoichiometry and high light response, also results in glucose sensitivity [38,39]. The involvement of Sll1252 in the electron transport from the PQ pool to the Cyt *b_6_/f* complex, like NDH complexes, might be the reason for the glucose sensitivity of Δ*sll1252^ins^*. However, the exact mechanism by which glucose exerted slow-growth phenotype under photo-mixotrophic and photo-heterotrophic conditions needs to be studied in detail.

**Figure 8:**
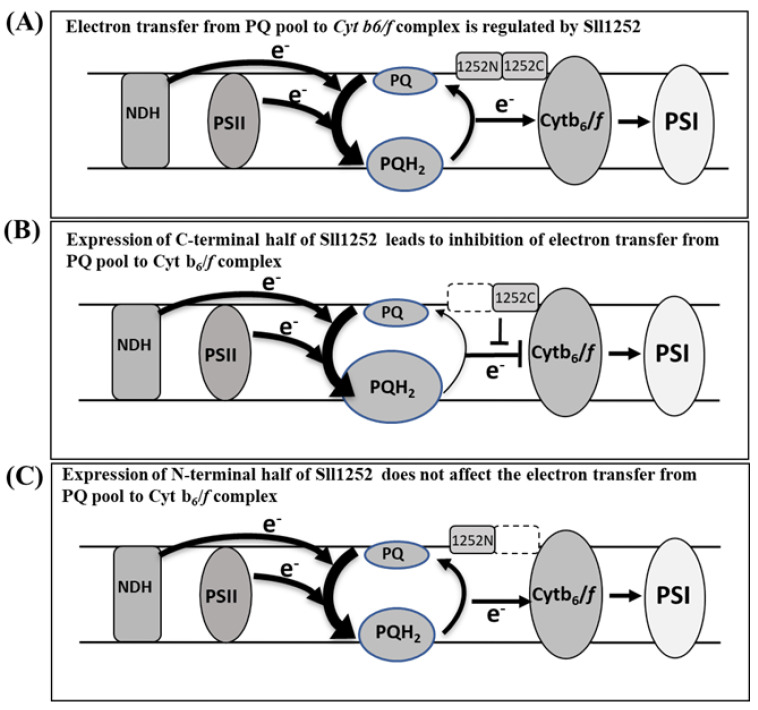
The model presented summarizes the function of Sll1252. (**A**) As the PQ pool is a common electron carrier and receives electrons from the NDH complex and PSII, the size of the PQH_2_ is represented with a bigger oval shape than the oxidized PQ. Sll1252 is probably associated with the Cyt *b_6_/f* complex. Electron transfer is regulated by the Sll1252 N- and C-terminal regions depending on the efficiency of the electron transport chain, thus balancing the functional coordination between PSII and PSI. For simplification, cyclic electron transport, terminal oxidases, and ATP synthase complexes are not shown. (**B**) Inhibition of electron transfer from the PQ pool to the Cyt *b_6_/f* complex due to the expression of the C-terminal region of Sll1252. The expression of the C-terminal leads to a further increase in the reduced-PQ pool. (**C**) Expression of the N-terminal region of Sll1252 has no effect on electron transfer between the PQ pool and the Cyt *b_6_/f* complex.

We have seen that the strain expressing only the C-terminal region of Sll1252 is detrimental to the cell, unlike the deletional mutant, in which Sll1252 is not expressed or the wild-type in which Sll1252 is expressed. The C-terminal region of Sll1252, consisting of the S4-domain, may act as an inhibitor of electron flow. The observations suggest that only N-terminal or full-length Sll1252 expression is not lethal to the cell. The lethality may have neutralized when both terminal proteins are expressed together. Thus, we propose that the interaction of the N-terminal region with the C-terminal region probably masks the inhibitory effect of the C-terminal region, thereby regulating the electron flow. Depending on the requirement, the electron transport rate may be regulated by Sll1252 using both its N-terminal and C-terminal regions. However, a detailed study is needed on the mechanism by which Sll1252 is involved in such a regulation. We present a model that shows the various experimental results as the basis for drawing a conclusion about the function of Sll1252 in the thylakoid membrane (Figure 8). Being associated with purified PSII complexes [6], this protein is assumed to have a transmembrane domain. However, computational analysis of the protein indicated that it does not possess any predicted transmembrane domains (Figure 1). Sll1252 may be associated with the thylakoid membrane in close vicinity of the PSII complex and may not be a direct member, but it is involved in efficient electron transfer from the reduced-PQ pool to the Cyt *b_6_/f* complex (Figure 8A). Expression of only the C-terminal region of Sll1252 leads to inhibition of electron transfer between the PQ pool and the Cyt *b_6_/f* complex (Figure 8B). Expression of only the N-terminal region of Sll1252 has no effect on electron transfer (Figure 8C). From the data obtained using *sll1252* truncated mutants, we suggest that the C-terminal region of Sll1252 down-regulates the electron flow. A kind of dynamic conformational change in Sll1252 probably leads to the specific interaction of the C-terminal region with the Cyt *b_6_/f* complex and is the key to the regulation of electron transport.

## Figures and Tables

**Table 1:** Comparison of photosynthetic activity and growth of Δ*sll1252* strains with the wild-type. Photosystem II and whole chain electron transport activities of wild-type and Δ*sll1252* strains, which were grown at 34 °C and bubbled with filter-sterilized air and 70 µmol photon m^−2^ s^−1^. Data represent the average of three independent experiments with standard deviations.

Strain	Mean Generation Time (h)	PSII Activity (H_2_O to PBQ)	WCE Activity (H_2_O to MV)
Wild-type	8.3 ± 0.3	1 ± 0.11	1 ± 0.13
Δ*sll1252^ins^*	Not detected	0.7 ± 0.11	0.55 ± 0.06
Δ*sll1252^del^*	9.8 ± 0.2	0.78 ± 0.23	0.82 ± 0.06
Δ*sll1252^inv^-Rev*	8.1 ± 0.2	0.89 ± 0.16	0.91 ± 0.09
Δ*sll1252-N^trn^*	22.0 ± 2.5	0.75 ± 0.04	0.55 ± 0.06
Δ*sll1252-C^trn^*	11.5 ± 0.7	0.59 ± 0.03	0.91 ± 0.06

**Table 2:** Genes whose expression were significantly affected due to the mutation in Δ*sll1252^ins.^*.

ORF and Description	(Δ*sll1252*^ins^/Wild-Type)		Effect of Inhibitor on Expression ^a^	
	5 µmol/m^−2^ s^−1^	70 µmol/m^−2^ s^−1^	DBMIB	DCMU
sll0330, sepiapterine reductase	1.3 ± 0.4	18.7 ± 5.5		
slr1544	1.1 ± 0.1	17.8 ± 1.7	I	
slr1704	1.3 ± 0.1	11.9 ± 0.2		
slr1674	1.1 ± 0.1	8.3 ± 0.6	I	
sll0528	1.1 ± 0.1	7.6 ± 0.2	I	
sll1483, periplasmic protein	1.1 ± 0.0	7.2 ± 0.7	I	I
sll1652	1.2 ± 0.1	6.6 ± 3.6		
sll0306, *sigB*, *rpoD*, RNA polymerase group 2 sigma factor	1.5 ± 0.1	6.5 ± 0.4	I	
sll1862	1.2 ± 0.1	5.8 ± 0.8		
sll1515, *gifB*, glutamine synthetase inactivating factor IF17	1.2 ± 0.1	5.6 ± 1.0	I	I
sll1653 *menG*, probable phylloquinone biosynthesis methlytransferase	0.9 ± 0.1	5.5 ± 0.9		
sll1514, *hspA*, *hsp1*, 16.6 kDa small heat shock protein	1.1 ± 0.1	5.5 ± 1.3	I	
ssr2194	1.1 ± 0.1	5.2 ± 0.1		
slr1516, *sodB*, superoxide dismutase	0.8 ± 0.1	4.9 ± 0.4		
sll1236	1.0 ± 0.1	4.9 ± 0.0		
slr1738	1.1 ± 0.1	4.7 ± 0.2	I	
slr0581	1.0 ± 0.1	4.5 ± 0.5	I	I
ssr2016	1.1 ± 0.0	4.1 ± 0.2		
slr2075, *groES*, *10 kDa chaperonin*, *GroES* protein	0.7 ± 0.0	3.9 ± 0.5	I	
ssr2595, *hilB*, *scpD*, high light-inducible protein	1.1 ± 0.1	3.9 ± 0.2	I	I
sll1598, *mntC*, ABC-type manganese transport system substrate-binding protein	1.1 ± 0.2	3.7 ± 0.8		
slr1675, *hypA1*, putative hydrogenase expression/formation protein HypA1	0.7 ± 0.1	3.7 ± 0.5	I	
slr2076, *groEL1*, *cpn60*, 60 kDa chaperonin 1, GroEL1, molecular chaperone	0.9 ± 0.0	3.7 ± 0.1	I	R
slr0967	0.8 ± 0.2	3.6 ± 0.4	I	I
ssl3044, hydrogenase component	1.0 ± 0.1	3.5 ± 0.4		
sl0416, *groEL2*, *cpn 60*, 60 kDa chaperonin 2, *GroEL2*,	1.1 ± 0.0	3.5 ± 1.1		R
sll0846	1.0 ± 0.0	3.2 ± 0.3	I	
sll1863	1.0 ± 0.1	3.2 ± 1.3		
slr0093, *dnaJ*, *DnaJ* protein, heat shock protein 40, molecular chaperone	1.1 ± 0.0	3.1 ± 0.2	I	
ssl1911, *gifA*, glutamine synthetase inactivating factor IF7	1.3 ± 0.1	2.9 ± 0.5	I	I
slr1687	1.1 ± 0.2	2.8 ± 0.1	I	I
sll1620	1.2 ± 0.0	2.8 ± 0.6	I	
slr1204, *htrA*, serine protease Htra	1.1 ± 0.2	2.8 ± 1.2		
sll1621, membrane protein	0.8 ± 0.1	2.7 ± 0.4	I	
slr1963, water-soluble carotenoid protein	1.1 ± 0.1	2.6 ± 0.8	I	R
ssl3364, *cp12*	1.3 ± 0.0	2.6 ± 0.7		
ssl2542, *hliA*, *scpC*, high light-inducible protein	0.8 ± 0.1	2.6 ± 0.5	I	

Wild-type and Δ*sll1252^ins^* cells were grown at 34 °C for 48 h, at an illumination of 70 µmol photons m^−2^ s^−1^ or at 5 µmol photons m^−2^ s^−1^. Each value indicates the ratio of levels of the mRNA from Δ*sll1252^ins^* cells to those from wild-type cells. The values shown are the means ± range of two independent experiments. The numbering of ORFs corresponds to that of Kaneko et al. [34]. ^a^ DBMIB, the effect of DBMIB, and DCMU, the effect of DCMU, on the expression of genes, according to Hihara et al. [18]. “I” denotes induction of the gene and “R” denotes repression of the gene.

## Data Availability

The data supporting the findings of this study are available within the article and Supplementary Materials.

## Supplementary Materials

The following supporting information can be downloaded at: https://www.mdpi.com/article/10.3390/genes14122151/s1. Figure S1: The reduction rate of the PQ pool in the DBMIB-treated and -untreated wild-type cells and Δ*sll1252^ins^* mutant cells [40]. Figure S2: Comparison of DNA microarray hybridization signal intensities between two datasets. Table S1: Microarray data of wild-type and ∆*sll1252* under 70 µmol photons m^−2^ s^−1^ and 5 µmol photons m^−2^ s^−1^.

## Appendix A

**Table A1:** List of primers used to generate DNA constructs and sequencing reactions.

Sl. No	Primer Name	Sequence
1	*sll1252-F*	5′-AAC CAT AGG CGC ATT GTA GCT CCT TG-3′
2	*sll1252-R*	5′-GAT ATC AAC ATT ATC CTT TGC CTC GAA CG-3′
3	KAN-2 FP-1	5′-ACC TAC AAC AAA GCT CTC ATC AAC C-3′
4	KAN-2 RP-1	5′-GCA ATG TAA CAT CAG AGA TTT TGA G-3′
5	Supp-F	5′-AGA TCT GCC AAG ATA ATT GAT TG-3′
6	Supp-R	5′-AGA TCT GTC GAC ATT CCA CTG GCA AAA-3′
7	UF	5′-GCA TCA ATT TGT TCT GTC AC-3′
8	UR	5′-AGA TCT CGA GCA ACG GCA TTA GCT-3′
9	DF	5′-CGT TGC TCG AGA TCT CCA GTT TCA AGG CTT GCC-3′
10	DR	5′-CCA CAA TTA AAG TTG TAC TG-3′
11	KAN-2 SalI-F	5′-TCG TCG ACC AAC CAT CAT CGA TGA ATT-3′
12	KAN-2 SalI-R	5′-TCG TCG ACA AAG CCG CCG TCC CGT CAA-3′
13	*sll1252-trn*-UR	5′-CGA GCA ACG GCA TTA GCT AA-3′
14	*sll1252-N^trn^*-F	5′-TAA TGC CGT TGC TCG ATG GTG CGT ACT GTA-3′
15	*sll1252-N^trn^-R*	5′-CCC GGG TTA AAG ATA TCG GGT-3′
16	*sll1252-N^trn^-DF*	5′-TAT CTT TAA CCC GGG CCA GTT TCA AGG CTT-3′
17	*sll1252-trn*-DR	5′-CCC GGG TTA CTG GGT TAA ATG AAG ACT-3′
18	*sll1252-C^trn^*-F	5′-TAA CCC AGT AAC CCG GGC CAG TTT CAA GGC TT-3′
