# Supplementary material for: Sll1252 Coordinates Electron Transport between Plastoquinone and Cytochrome b6/f Complex in Synechocystis PCC 6803
Source: Genes (Basel). 2023 Nov 28;14(12):2151. doi: 10.3390/genes14122151 (PMC10743179; doi:10.3390/genes14122151)
Supplement: Supplementary file 1 [file genes-14-02151-s001.zip › Supplementary Material, Figure S2- MDPI-Genes-Sll1252 28 Nov 2023.pdf]

Supplementary Material

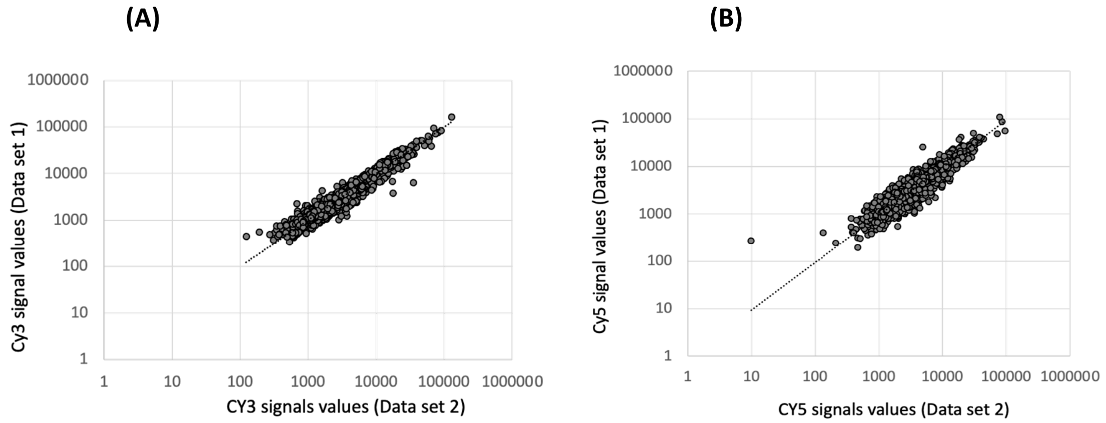

Figure S2: Comparison of DNA microarray hybridization signal intensities between two data sets. The wild type *Synechocystis* (WT) and Δ*sll1252* cells were grown for 48 h, at 70 µmol photons m^{-2} s^{-1}. cDNA synthesized from WT and Δ*sll1252* RNA were labelled with Cy3 and Cy5 fluorescence dyes respectively and hybridized to Cyano-chip ver. 1.6. The TIFF images were processed and, Cy3 and Cy5 signal intensities for each feature were converted into numerical signals. Both Cy3 and Cy5 signal values were globally normalized, and the fold changes were calculated for two data sets. (A) scatter plot of Cy3 signal values of all the genes from data set-1 versus Cy3 signal values of data set-2. (B) scatter plot of Cy5 signal values of two data sets. The line of best fit in both the cases passes through the origin signifying high correlation between two data sets.
