# Supplementary material for: Sll1252 Coordinates Electron Transport between Plastoquinone and Cytochrome b6/f Complex in Synechocystis PCC 6803
Source: Genes (Basel). 2023 Nov 28;14(12):2151. doi: 10.3390/genes14122151 (PMC10743179; doi:10.3390/genes14122151)
Supplement: Supplementary file 1 [file genes-14-02151-s001.zip › Supplemntary Material, Figure S1- 28 Nov 2023 .pdf]

Determination of the rate of PQ pool reduction

Method: The reduction rate of the PQ pool in the intact cells was determined by changes in absorption of a measuring light at 260 nm upon the initiation of irradiation of the actinic light at 600 nm at 70 μmole photons m^{-2} s^{-1}, following the method reported previously [35]. Briefly, the cells were grown at 30˚C for some days under constant illumination with incandescent lamps at 7 μmol photons m^{-2} s^{-1} with aeration of 2% CO_2-enriched air. The cells were suspended with fresh BG-11 medium at $O.D._{730}$ 1.5. The cell suspension was measured for PQ pool redox state in a spectrophotometer, UVIDEC-4 (JUSCO, Tokyo, Japan), installed in a dark room at 25˚C. The cell suspension was aerated for more than 5 min with air to maintain the redox state of the PQ pool at a certain level by the respiration under the O_2-sufficient conditions. During the preincubation period, absorption of 260 nm by the cell suspension became constant. The illumination and aeration were halted just before measurement and immediately initiated the illumination of the actinic light to monitor the initial rate of the change in the absorption at 260 nm.

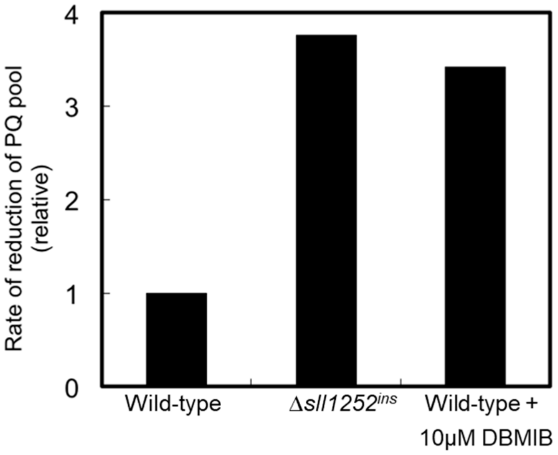

Figure S1: The reduction rate of the PQ pool in the DBMIB-treated and untreated wild-type cells and *Δsll1252*^{ins} mutant cells. The reduced plastoquinone rates were monitored spectroscopically by measuring OD at 260nm. Cells were incubated in the dark for one minute and then continuously exposed to actinic light (600 nm) for 1 minute. During the dark and light incubation, O.D. at 260 nm was monitored.

Result: In support of the experiments demonstrating the site of action of Sll1252 in this study, the mutation in *sll1252* impairs the transfer of electrons from the reduced PQ pool to Cyt b_6/*f* complex (Table 1). The consequence of this impaired rate of electron transfer would lead to a relatively higher level of plastoquinones in a reduced state in *Δsll1252*^{ins} cells grown at 70 μmol photons $m^{-2}s^{-1}$ when compared to wild-type cells that had been grown in light at the same intensity. Hence, the rate of PQ reduction was estimated. As shown in Figure S1, enhancement in the rate of PQ pool reduction was observed in Δ*sll1252*^{ins} mutant when compared to wild-type cells grown in light at 70 μmol photons m^{-2} s^{-1}. Relatively, a 4-fold increase in the PQ reduction rate was observed in Δ*sll1252*^{ins} compared to wild-type *Synechocystis* cells. In wild-type cells, inhibition of

electron transport by DBMIB also resulted in an enhanced rate of PQ reduction, consistent with the previous report by Kashino et al. [40].
